# Queer in Chem: Q&A with Professor Anna G. Slater

**DOI:** 10.1038/s42004-023-00974-7

**Published:** 2023-09-30

## Abstract

Anna G. Slater is a Professor of Chemistry and Royal Society University Research Fellow at the University of Liverpool’s Materials Innovation Factory and Chemistry Department. Exploiting continuous flow processes for enhanced control of chemistry is a central theme of her work, which spans molecular materials, supramolecular chemistry, and sustainable synthesis.

**Figure:**
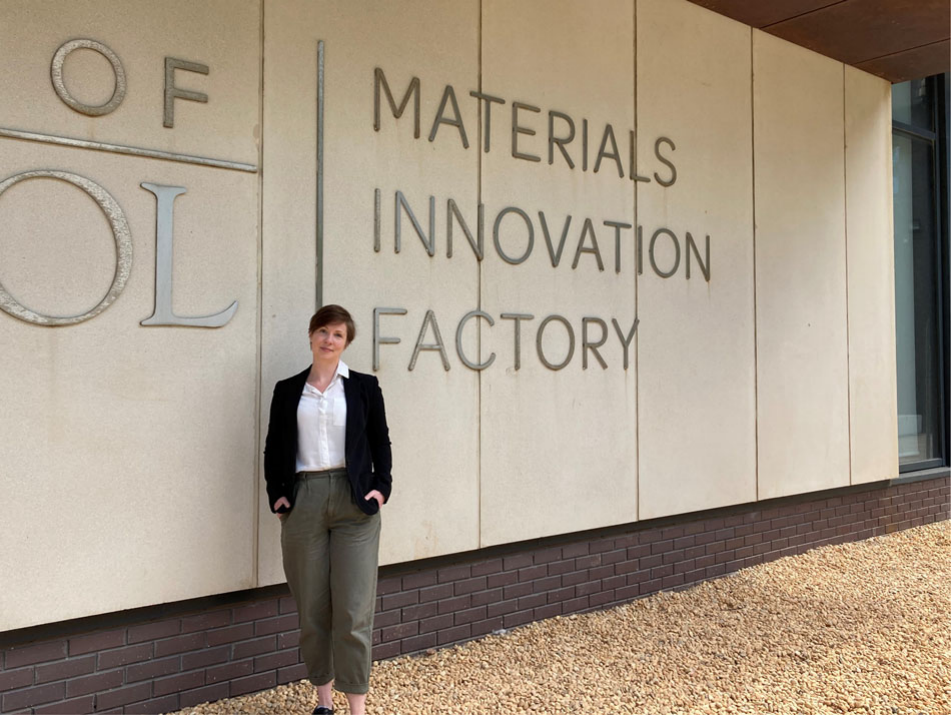
Anna G. Slater

Why did you choose to be a scientist?

I can’t remember actively choosing to be a scientist! When choosing my A-Levels I nearly put down English Language, Philosophy, and History— I could have ended up in a very different place— but in the end I picked Chemistry because of the pull of finding out how things work. I also had a role model in my dad, who is a scientist. He got his PhD while working part-time as an electron microscopist when I was in primary school, and went on to have a career in research working between academia and the National Health System. I didn’t necessarily plan to stay in science beyond my undergraduate degree, but the opportunities to solve problems, work with people with diverse perspectives, and have the creative freedom to work on interesting challenges has kept me here.

What scientific development are you currently most excited about?

I am very excited about the ways that developing technology is changing how we carry out scientific research. We can understand chemical processes in more depth, explore wider, and iterate faster than was possible even 20 years ago when I was an undergrad. It’s a very exciting time to be a chemist— and to think about the skills future chemists will need. In our lab, I’m very excited about early results that demonstrate that we can tune the outcome of reversible chemical processes by exerting better control over the environment those processes happen in. If we get this right, there are a lot of fun things we can do…

What direction do you think your research field should go in?

There is a pressing need to make scientific research more environmentally sustainable, more reproducible, and more accessible. I’m involved in a few fields: supramolecular chemistry^1,2^, flow chemistry^3–6^, and materials chemistry^7,8^. In each, automation, data processing, advanced analytical tools, and ‘digital chemistry’ are beginning to influence how we approach research. For me, the most important thing is to ensure that these advances don’t only benefit those who develop them, but are available and accessible to all researchers. We need to engage multidisciplinary teams to use and shape these methods to tackle global challenges such as the climate crisis, ensuring reach beyond ‘early adopters’.

How does your queer or trans identity intersect with your identity as a scientist?

My identities as a scientist and a queer person are not necessarily the strongest or most visible elements, so it is interesting to consider how they intersect. I am a bi/pan person in a relationship that most people read as heterosexual. Similarly, most people assume I identify as a woman, but I don’t feel a strong connection to gender (I am comfortable with any pronouns, mainly using she/her for convenience, and I can see why people assume!). People’s assumptions can have advantages—passing for ‘straight’ can bring safety, for example, I have only rarely experienced biphobia, and have never experienced discrimination or fear of violence for being queer. However, these assumptions can also be erasing and harmful.

Why do you think it is important to feel comfortable enough to bring your whole self to work?

This is an interesting question. I’m not sure I do bring my whole self to work—and this means like anyone else in this situation I have a degree of mental load from ‘masking’ aspects of myself or my experience. For me, this happens more frequently in the context of being a disabled scientist. I can’t always choose whether it comes up at work, and when it does, I have to decide whether to disclose, explain why I can’t do something or need adjustments, or to correct assumptions. There is always a worry that people will respond negatively or that it will have a negative impact on me or our team.

If we remove this worry and ensure a supportive environment where people do feel comfortable to bring their whole selves to work, we allow people to thrive. They can spend time and energy on their research rather than suppressing parts of themselves to get by. This takes a lot of hard work and education though, and a willingness to shift the research culture; here, supportive external networks can be critical. I’ve benefited enormously from being part of WISC (Women in Supramolecular Chemistry—a network which aims to support women and all marginalized groups);^9–12^ a space where I can be more of my ‘full self’. The conversations that can be had—and the explanations that don’t need to happen—are liberating.

How can individual scientists support and celebrate their LGBTQ + colleagues?

Firstly, recognize that one approach to support and celebration will not suit everyone who identifies as LGBTQ +; it may be best to ask your community to find out what your colleagues want and need.

Avoid making assumptions and be aware of the challenges queer and trans scientists face. For concrete steps to take, the Royal Society of Chemistry LGBT+ toolkit is a great place to start: [HYPERLINK: https://www.rsc.org/policy-evidence-campaigns/inclusion-diversity/resources/lgbt-toolkit/].

We can also examine our own privileges. Recognizing where we have privileges does not mean erasing where we have faced challenges. For example, if I think about my own privilege, my dad is a scientist and was an academic; this can give me advantages such as familiarity with the academic context. I am white and thus not marginalized or discriminated against due to my race and ethnicity. I can also ‘pass’ or hide my sexuality, gender identity, and disability, and I’m now a more senior scientist than I used to be! All these together can give me access to spaces where I can influence decisions. I must recognize that and ensure I am (a) making space for others; (b) advocating for equity; (c) using this (often unearned) power for positive change.

We must listen to those with lived experience of marginalization and ensure their voices are central in any plans for support. We must also listen to those who have expertise in how supportive cultures are built: I’m a chemist; I’m not an expert in equity, diversity, and inclusion. We all need to recognize when our expertise isn’t enough, when we don’t know the literature, the research, or the context. In these situations, we need to pay, fund, and collaborate with experts alongside educating ourselves with the huge range of resources that are out there.

*This interview was conducted by the editors of Communications Chemistry*.
